# Systemic steroids in COPD-the beauty and the beast

**DOI:** 10.1186/1465-9921-15-38

**Published:** 2014-04-03

**Authors:** Claus F Vogelmeier

**Affiliations:** 1 Department of Medicine, Pulmonary and Critical Care Medicine, 35033 Marburg, Germany; 2 Member of the German Center for Lung Research (DZL), University Medical Center Giessen and Marburg, Philipps-Universität Marburg, 35033 Marburg, Germany

## 

The recommendations regarding the use of systemic steroids in COPD differ substantially depending on the phase of the disease. In moderate and severe acute exacerbations oral steroids are advocated based on the findings of several placebo-controlled trials that have been performed in secondary-care settings: these studies showed that systemic steroids improve lung function, dyspnoea and gas exchange. In addition, steroid use resulted in fewer treatment failures, a lower relapse rate and shorter hospital stays [1-4]. Importantly, giving steroids orally is non-inferior to the i.v. application [5] and an 8-week treatment is not superior to a 2-week therapy [6]. Recently, it could even be shown that 5-day treatment is non-inferior to a 14-day course [7]. In the trial with longer term therapy [6] hyperglycemia requiring treatment was identified as considerable side effect, in the other trials no major adverse events were observed.

In contrast, in stable COPD positive effects of systemic steroids are scarce: there is some evidence that higher doses (≥ 30 mg prednisolone/day) may improve lung function over a short period, whereas this was not observed in doses of less than 10–15 mg prednisolone/day [8]. In a 2-year trial with 5 mg prednisolone + 1600 μg budesonide/day efficacy regarding lung function decline was not significantly better than budesonide alone [9]. In addition, there are substantial side effect problems. In particular, it is well established that respiratory and peripheral muscle strength [10] are affected. This is caused by a myopathy that is characterized by atrophy and necrosis [11] and may lead to reduced survival. Besides, there is evidence that chronic therapy with systemic steroids is one of the risk factors for the high rate of osteoporosis that is observed in COPD [12].

What does the paper by Horita et al. published in this issue of the journal [13] add to the literature? To my knowledge this is the first analysis that systematically evaluates the effects of systemic steroids on mortality in a considerable sample of COPD patients with severe and very severe COPD. The authors analyzed patients (N = 444) that had been randomized to the conservative treatment arm in the National Emphysema Treatment trial (NETT). They calculated a hazard ratio for death of 1.54 for the steroid user group. Then, in order to make sure that this result was not induced by unidentified confounders they performed a propensity score matching. Based on this analysis the hazard ratio for death in the steroid group was 1.73. Thus, the observed effect seems to be robust and real.

Nevertheless, the authors righteously mention several potential weaknesses of their study: a) it is not prospective, but a prospective trial with this endpoint does not seem feasible, b) the steroid dose was not verified, c) only patients with an FEV1 ≤ 45% predicted had been included in the NETT trial, but logic tells that systemic steroids should only be considered when all other therapies are not sufficiently effective and it is quite unlikely that this occurs in patients with a moderate lung function impairment. It is quite worrisome though that in the NETT trial only a minority of the patients were treated with long acting bronchodilators.

In summary, the study results support the recommendations in guidelines and strategy documents to stay away from the chronic use of systemic steroids in COPD [14]. As a consequence, in patients in stable condition that are currently treated with systemic steroids this therapy should be tapered/stopped. This can be quite demanding: patients may develop fatigue, low blood pressure, joint pain, weakness and even psychotic symptoms [15]. Importantly, discontinuation of chronic systemic steroid therapy did not result in an increase of COPD exacerbations [16]. There is only one scenario where steroid cessation may be harmful: some patients with an asthma-COPD overlap may profit from long-term use of systemic steroids [17] and may get worse when steroids are stopped.

Based on the cited literature and supported by the findings of Horita et al. [13] there are some simple messages regarding the use of systemic steroids in COPD: a) in moderate or severe acute exacerbations oral steroids should be prescribed in a moderate dose (40 mg prednisolone) for 5 days, b) there is no place for systemic steroids in stable phase COPD, c) in patients treated with systemic steroids outside of an exacerbation steroid treatment should be withdrawn, d) before doing so therapy should be optimized and patients should be re-evaluated to make sure that they do not have an asthma-COPD overlap.
